# The origin of modern frogs (Neobatrachia) was accompanied by acceleration in mitochondrial and nuclear substitution rates

**DOI:** 10.1186/1471-2164-13-626

**Published:** 2012-11-15

**Authors:** Iker Irisarri, Diego San Mauro, Federico Abascal, Annemarie Ohler, Miguel Vences, Rafael Zardoya

**Affiliations:** 1 Department of Biodiversity and Evolutionary Biology, Museo Nacional de Ciencias Naturales, CSIC, José Gutiérrez Abascal 2, 28006, Madrid, Spain; 2 Department of Animal Biology, University of Barcelona, Av. Diagonal 643, 08028, Barcelona, Spain; 3 Structural Computational Biology Group, Spanish National Cancer Research Center (CNIO), Melchor Fernández Almagro 3, 28029, Madrid, Spain; 4 UMR 72205 OSEB, Département de Systématique et Evolution, Muséum National d’Histoire Naturelle, 25 rue Cuvier, CP 30, 75005, Paris, France; 5 Division of Evolutionary Biology, Zoological Institute, Technical University of Braunschweig, Mendelssohnstrasse 8, 38106, Braunschweig, Germany

**Keywords:** Substitution rate, Selection, Molecular dating, Mitochondrial genome, Mitogenomics, Anura, Neobatrachia, Evolution

## Abstract

**Background:**

Understanding the causes underlying heterogeneity of molecular evolutionary rates among lineages is a long-standing and central question in evolutionary biology. Although several earlier studies showed that modern frogs (Neobatrachia) experienced an acceleration of mitochondrial gene substitution rates compared to non-neobatrachian relatives, no further characterization of this phenomenon was attempted. To gain new insights on this topic, we sequenced the complete mitochondrial genomes and nine nuclear loci of one pelobatoid (*Pelodytes punctatus*) and five neobatrachians, *Heleophryne regis* (Heleophrynidae)*, Lechriodus melanopyga* (Limnodynastidae)*, Calyptocephalella gayi* (Calyptocephalellidae)*, Telmatobius bolivianus* (Ceratophryidae)*,* and *Sooglossus thomasseti* (Sooglossidae). These represent major clades not included in previous mitogenomic analyses, and most of them are remarkably species-poor compared to other neobatrachians.

**Results:**

We reconstructed a fully resolved and robust phylogeny of extant frogs based on the new mitochondrial and nuclear sequence data, and dated major cladogenetic events. The reconstructed tree recovered *Heleophryne* as sister group to all other neobatrachians, the Australasian *Lechriodus* and the South American *Calyptocephalella* formed a clade that was the sister group to Nobleobatrachia, and the Seychellois *Sooglossus* was recovered as the sister group of Ranoides. We used relative-rate tests and direct comparison of branch lengths from mitochondrial and nuclear-based trees to demonstrate that both mitochondrial and nuclear evolutionary rates are significantly higher in all neobatrachians compared to their non-neobatrachian relatives, and that such rate acceleration started at the origin of Neobatrachia.

**Conclusions:**

Through the analysis of the selection coefficient (ω) in different branches of the tree, we found compelling evidence of relaxation of purifying selection in neobatrachians, which could (at least in part) explain the observed higher mitochondrial and nuclear substitution rates in this clade. Our analyses allowed us to discard that changes in substitution rates could be correlated with increased mitochondrial genome rearrangement or diversification rates observed in different lineages of neobatrachians.

## Background

It has been long acknowledged that character change in evolution occurs at different rates, which can vary extremely between different lineages [1]. Although initially described for morphological characters, among-lineage rate heterogeneity also occurs at the molecular level, and evolutionary biologists have been long interested in quantifying molecular evolutionary rates as well as determining which are the underlying mechanisms that trigger their acceleration or slowdown in different lineages [2]. However, uncovering the causes of lineage-specific rate variation has proven to be challenging, and previous studies reached different conclusions, which attempted to explain rate heterogeneity through correlation with species body size, generation time, population dynamics, metabolic rates, or habits (*e.g.*, [2-4]). Furthermore, molecular evolutionary rates have also been correlated with diversification [5-7], but given the multiple factors that shape diversification patterns, the generalization of this correlation is elusive, and therefore, the cause-effect between rates of genome evolution and cladogenesis remain largely unknown [2].

At the molecular level, the fixation of mutations in an evolutionary lineage (*i.e.,* substitution events), is a complex dynamic process determined by the interaction between evolutionary (selection) and demographic (drift) forces [8]. Comparative studies of relative substitution rates have been particularly useful in providing insights into particular constraints of specific genetic systems, such as *e.g.* mitochondrial (mt) genomes [9]. Mitochondrial DNA has been widely used as a marker in molecular systematics during past decades [10,11]. As data accumulated, it has become apparent that animal mt DNA evolves at a rate 5 to 10 times faster than single-copy protein coding nuclear genes, although this varies extremely across genes and taxa [10,12,13]. Mitochondrial DNA suffers from high mutational pressure [14] likely due to the inaccuracy of its DNA repair system [15], the absence of histone-like proteins [14], the particular replication model with single-strand intermediates [16], and the presence of reactive oxidative compounds produced in the mitochondria [17]. This high mutational pressure, together with a reduced population size [18] and the absence of substantial recombination [19] (but see [20]), leads to an increase of substitution rate in mt DNA. The comparison of evolutionary rates among lineages permits the identification of events of acceleration and slowdown of rates, which can be further studied to uncover the underlying process (or processes) that produced the observed patterns [2].

Furthermore, phylogeneticists are particularly interested in understanding evolutionary rate variation because the unequal substitution rates among lineages are a well-known source of phylogenetic artifacts [21,22]. Rapidly evolving lineages may appear closely related (and often placed close to outgroups) regardless of their true evolutionary relationships (long-branch attraction; [23]), whereas short branches may also attract each other because of the “leftover” similarity of symplesiomorphic states that “eroded” away in rapid-evolving lineages [24].

Previous studies have shown that rates of molecular evolution, both for mt DNA and some nuclear genes are unequally distributed among lineages of frogs [25]. Neobatrachian frogs exhibit higher mt substitution rates compared to their non-neobatrachian relatives [25-29]. Yet, it is neither clear when the shifts in substitution rates occurred during the evolutionary history of modern frogs nor whether rate changes are exclusive to the mt genome. Moreover, the heterogeneous distribution of mt substitution rates, together with the high genetic divergence between frogs and their closest living sister taxa (*i.e.,* salamanders; [30]) are the source of several phylogenetic artifacts in previous studies, such as the monophyly of non-neobatrachian frogs (“Archaeobatrachia”: [29,31,32]) or the incorrect phylogenetic placement of Neobatrachia due to long-branch attraction effects [27]. The unequal distribution of mt substitution rates across the anuran tree has also been suggested to yield considerably older time estimates for divergences among neobatrachians [33].

Neobatrachia (modern frogs) is acknowledged as the most derived lineage of frogs [34,35] and the sister group of Pelobatoidea [36,37]. Modern frogs constitute an evolutionarily highly successful clade that contains over 96% of the overall species diversity of extant amphibians [38-40]. Most of this diversity is concentrated in two major clades: Ranoides (= Ranoidea), which comprises three well-supported monophyletic groups (Afrobatrachia, Microhyloidea, Natatanura), and Nobleobatrachia [39,41]. Neobatrachia` also includes the following species-poor families: Calyptocephalellidae, Heleophrynidae, Limnodynastidae, Myobatrachidae, Nasikabatrachidae, and Sooglossidae, whose relative position is still a contentious issue in anuran phylogeny [39,41]. Most of the diversity of Ranoides and Nobleobatrachia is located in the Old World and the Neotropics, respectively [25], whereas the abovementioned species-poor neobatrachian families show a relict distribution [40]. It was suggested that the shift in mt substitution rates in Neobatrachia could be related with the higher diversification rates observed in Ranoides and Nobleobatrachia, provided that further data and analyses could possibly assign short branches to species-poor lineages [25]. To validate such possibility, it is necessary to precisely delimit the node in the anuran phylogeny at which the shift in evolutionary rates took place.

In this study, we newly determined the mt genomes and partial sequences of nine nuclear genes of several key representatives of species-poor neobatrachian families outside Ranoides and Nobleobatrachia. These new sequence data together with previously available orthologous sequence data from other anurans were used to infer a robust phylogeny and a timetree of major lineages of frogs. The new sequence data and the phylogeny were used to (*i*) confirm that mt substitution rates are (statistically) significantly higher in the different neobatrachian lineages; (*ii*) localize the rate shift in the phylogeny; (*iii*) explore whether substitution rates of nuclear genes are also accelerated in modern frogs; and (*iv*) determine whether changes in substitution rates could be correlated with life-history traits, an increase in mt genome rearrangement, or an increase in diversification rates.

## Methods

### Taxon sampling and DNA sequencing

Taxon sampling in this study was designed to represent all major groups of frogs with particular emphasis on neobatrachian lineages outside Ranoides and Nobleobatrachia. For phylogeny reconstruction, we used *Leiopelma* and *Ascaphus* as outgroup taxa. For timetree inference, the outgroup included three salamanders, three caecilians, a lizard, a bird, and a mammal. These extra outgroup taxa were included to provide additional calibration points, and thus to infer more accurate timetree estimates. In-group frog species were largely chosen based on available complete mt genomes, and to allow direct comparison and combination of mt and nuclear data. Available frog mt genomes were expanded with newly determined complete sequences for one pelobatoid (*Pelodytes* sp., from the southwest of the Iberian Peninsula, probably representing an undescribed species currently under study; in this paper, we name it *P. punctatus* following current taxonomy) and the following neobatrachians: *Heleophryne regis* (Heleophrynidae)*, Lechriodus melanopyga* (Limnodynastidae)*, Calyptocephalella gayi* (Calyptocephalellidae)*, Telmatobius bolivianus* (Ceratophryidae)*, Sooglossus thomasseti* (Sooglossidae). The nearly complete sequence of another Sooglossidae, *Sooglossus sechellensis*, was also determined. A nuclear DNA data set of partial sequences of nine protein-coding genes was compiled, including the recombination-activating genes 1 and 2 (*rag1* and *rag2*), brain-derived neutrotrophic factor (*bdnf*), proopiomelanocortin (*pomc*), chemokine receptor type 4 (*cxcr4*, exon 2), members 1 and 3 of the solute carrier family 8 (*slc8a1,* exon 2, and *slc8a3*, exon2), rhodopsin (*rho* exon 1), and histone 3 (*h3a*). The nuclear matrix was generated expanding a recent dataset [37] with new data for the aforementioned species. Since our phylogenetic analyses were focused mainly on the family level or above, and in order to maximize the completeness of the nuclear data set, sequences from congeneric anuran species (for which there is strong evidence for the monophyly of the genus) were merged in few cases see (*e.g.,*[42-44]). Similarly, chimerical sequences were also used to represent some non-anuran major evolutionary lineages in the outgroup of the timetree analysis. Detailed information on the studied species and the corresponding GenBank accession numbers can be found in Additional file 1.

Total DNA was prepared from ethanol-preserved muscle tissue by proteinase k digestion, phenol-chloroform extraction, and ethanol precipitation [45]. The complete mt genome of *Pelodytes* was amplified in several overlapping fragments by PCR using the primers and conditions reported in [46]. The remaining mt genomes, corresponding to neobatrachians, were partially amplified using the same set of primers (from 5’-*trnF* to 3’-*cox3*) (abbreviations of mt genes follow [47]). Due to the gene rearrangements found in neobatrachians (see Results and discussion), the remaining halves of the mt genomes (from 5’-*cox3* to 3’-*trnF*) were amplified using the primers and conditions reported in [48]. Partial sequences of nuclear genes were amplified using the primers and conditions reported in the literature: *rag1*[46], *rag2*[25,49], *slc8a1*[36], *bdnf* and *pomc*[50], *rho*[25], *h3a*[51]. In all cases, PCR cycling conditions were experimentally adjusted from those reported in the original publications. Specific primers were designed when general primers did not work (mainly for control regions), and to sequence long-PCR products by primer walking (available from authors upon request). PCR reactions of fragments up to 1500 bp were carried out with 5PRIME *Taq* DNA polymerase (5PRIME GmbH, Hamburg, Germany), and longer fragments were amplified using LA *Taq* polymerase (TaKaRa Bio Inc., Otsu, Shiga, Japan), following manufacturer’s instructions. PCR amplicons were purified by ethanol precipitation [45] or directly from electrophoresis gels using the Speedtools PCR clean-up kit (Biotools B&M Labs. S.A., Madrid, Spain). The long-PCR products containing the control region of *L. melanopyga* and *T. bolivianus* were digested with *Pst*I at 37°C for four hours, obtaining two fragments from each of the original amplicons. These four fragments as well as all other PCR products containing the control regions of the remaining species were cloned into pGEM-T vectors (Promega, Madison, WI, USA). PCR fragments and positive recombinant clones were cycle-sequenced with the ABI Prism BigDye Terminator v3.1 cycle sequencing ready reaction kit (Applied Biosystems, Foster City, CA, USA) using PCR and M13 universal primers, and following manufacturer’s instructions. Cycle sequencing products were run on ABI Prism 3700 and 3130xl DNA Analyzers (Applied Biosystems, Foster City, CA, USA).

The new mt sequences were annotated by comparison with other reported frog mt genomes using DOGMA [52]. In this web-based tool, genes are identified by BLAST searches, open reading frames of protein-coding genes are translated using the appropriate genetic code (vertebrate mt code), and transfer RNA (tRNA) genes are further identified based on their putative cloverleaf secondary structure. The gene arrangements of the new mt genomes were compared against the Mitozoa database release 7.1 [53].

### Sequence alignment

Mitochondrial and nuclear protein-coding genes were analyzed at the nucleotide and amino acid levels. Alignments were generated using TranslatorX [54]. First, amino acids of deduced proteins were aligned using MAFFT L-INS-i [55] and default settings. Then, ambiguously aligned positions were removed using Gblocks, v.0.19b [56] and the following settings: minimum number of sequences for a conserved position 29, minimum number of sequences for a flanking position 29, maximum number of contiguous non-conserved positions 8, minimum length of an initial block 5, minimum length of a block 5, allowed gap positions with half. Finally, trimmed protein alignments were used as guide for a codon-based alignment of nucleotide sequences. Mitochondrial tRNA gene nucleotide sequences were aligned manually based on their putative secondary structure, whereas mt ribosomal RNA (rRNA) gene nucleotide sequences were aligned with MAFFT L-INS-i [55] and corrected by eye for any obvious misalignment. Ambiguously aligned positions in both mt tRNA and rRNA gene alignments were excluded with Gblocks v.0.19b [56] using the following settings: minimum number of sequences for a conserved position 31, minimum number of sequences for a flanking position 36, maximum number of contiguous non-conserved positions 5, minimum length of a block 10, allowed gap positions with half.

### Phylogenetic reconstruction

Previous studies (*e.g.,*[27]) showed long-branch attraction artifacts in phylogenetic reconstruction of the anuran tree due to high rates of evolution of mt DNA in neobatrachians. In order to avoid such phylogenetic inference artifacts, especially those caused by possible saturation in the fast-evolving lineages, first and third codon positions of mt protein-coding genes, and third codon positions of nuclear protein-coding genes were excluded from the corresponding nucleotide alignments. For phylogenetic and dating analyses, protein-coding gene alignments at the nucleotide or amino acid level were combined together with tRNA and rRNA genes into two matrices, hereafter the combined nucleotide and amino acid data sets*,* respectively.

The combined nucleotide and amino acid data sets were analyzed by maximum likelihood (ML; [57]) using RAxML v.7.0.4 [58], and by Bayesian inference (BI; [59]) using MrBayes v.3.1.2 [60,61]. ML searches used the rapid hill-climbing algorithm [62] starting from 100 randomized maximum-parsimony trees. RAxML optimized parameters of the GTR+I+Γ model in all partitions independently. For BI, two independent runs were performed, each with 4 simultaneous Markov chains for 20 million generations, sampling every 1000 generations. Convergence was checked *a posteriori* using Tracer v.1.5 [63] and the online tool AWTY [64]. The first 1 million generations were discarded as burn-in to prevent sampling before the Markov chains reached stationarity. Support for internal branches was evaluated performing 1000 replicates of non-parametric bootstrapping [65] (ML) and by posterior probabilities (BI). The genera *Leiopelma* and *Ascaphus* were used as outgroups, as they are confidently identified as the sister taxa of all other extant frogs [26,39,41,66,67].

The Akaike information criterion (AIC; [68]) was used to select the best partition schemes as well as best-fit nucleotide and amino acid models for each partition. The best partitioning scheme for the combined nucleotide data set was determined with PartitionFinder [69] and included five partitions: (*i*) second codon positions of all mt protein-coding genes, (*ii*) mt rRNA genes, (*iii*) mt tRNA genes, (*iv*) first codon positions of all nuclear genes, and (*v*) second codon positions of all nuclear genes. For the combined amino acid data set, partitions had to be determined separately for amino acid (PartitionFinderProtein; [69]) and nucleotide (PartitionFinder) data. The best partition scheme favored individual protein-coding gene partitions, a single mt rRNA partition and a single mt tRNA gene partition.

### Estimation of divergence times

**Figure 1:**
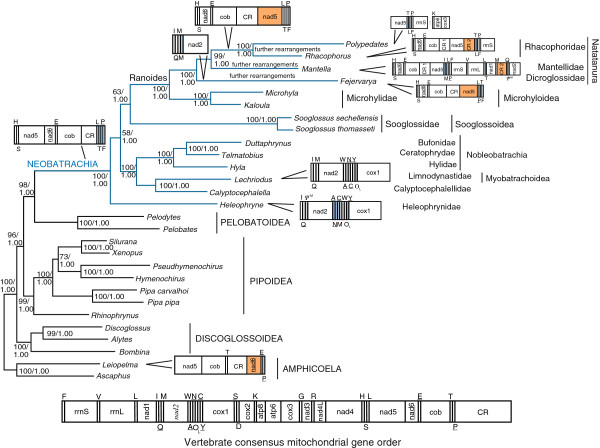
**Phylogenetic relationships of extant frogs.** The ML phylogram inferred from the combined nucleotide data set is shown. The inferred tree based on the combined amino acid data set arrived at the same topology, which represents our best hypothesis for anuran phylogenetic relationships. Numbers at nodes are support values from ML (bootstrap proportions; left) and BI (posterior probabilities; right). Names of major clades of frogs are shown in capitals, Neobatrachia is highlighted in blue, and familial and supra-familial assignments are indicated for neobatrachians. Scale bar is substitutions/ site. The consensus gene order of the vertebrate mt genome is shown as well as variations of this gene order along the phylogeny. Genes encoded by the light strand are underlined. Different colors are used to indicate the origin of replication of the light strand (grey), translocated protein-coding genes (orange) and transfer RNA genes (blue). Abbreviations of mt genes follow [47].

We used BEAST v.1.6.1 [70] to estimate divergence times among major frog lineages based on molecular data. This program implements a Bayesian dating method, and assumes a relaxed uncorrelated clock in which the rate for each branch is drawn independently from an underlying lognormal distribution [71]. We used the combined nucleotide data set*,* and constrained the tree topology to the best ML tree (Figure 1) by removing the operators that act on tree topology from the .xml file. The Yule process [72] was used to describe cladogenesis, and independent GTR+I+Γ models were applied for each of the five data partitions. The final Markov chain was run twice for 100 million generations, sampling every 10,000 generations and the first 1 million was discarded as part of the burn-in process, according to the convergence of chains checked with Tracer v.1.5. [63]. The effective sample size of all the parameters was above 200 [70].

Seven calibration points were used as priors for divergence times of certain splits, using a lognormal distribution of prior probability. Calibration points were chosen based on previous literature and the online resource Lisanfos KMS v.1.2 [73] that compiles data on amphibian fossils. Fossils provided hard minimum bounds (offset) and mean and standard deviations (SD) were chosen so that the 95% probability limit corresponds to a soft maximum bound. Details on fossil dates and prior distribution parameters for each calibration point are provided in Additional file 2.

### Evolutionary rate heterogeneity analyses

#### Topological congruence

To examine among-lineage rate heterogeneity patterns, we reconstructed phylogenetic trees based on mt and nuclear data, separately, and compared their topological congruence and branch length patterns. In these comparative analyses, we were not interested in recovering the species phylogeny (as above) but in evidencing the empirical differences in evolutionary rates between neobatrachian and non-neobatrachian lineages as inferred based on the two types of molecular markers. Therefore, all codon positions of protein-coding genes were included in the analyzed nucleotide data matrices.

Phylogenetic analyses were based on the concatenation of the nucleotide sequences of all mt single-gene alignments (hereafter the mt nucleotide data set) or all nuclear single-gene alignments (hereafter the nuclear nucleotide data set), as well as on the same alignments but with open reading frames translated into proteins (hereafter the mt amino acid and the nuclear amino acid data sets). Trees were reconstructed under ML as explained in the phylogenetic reconstruction section above. Protein-coding genes were partitioned by codon position in the mt and nuclear nucleotide data sets, or by gene in the mt and nuclear amino acid data sets. The mt nucleotide and amino acid data sets had two additional partitions corresponding to combined rRNA and tRNA genes, respectively.

#### Relative-rate tests

In order to compare substitution rates of mt and nuclear genes, relative-rate tests (RRTs; [74]) were performed with the program RRTree [75]. This program extends the method of Li and Bousquet [76] and compares mean rates between lineages relative to the outgroup, taking phylogenetic relationships into account by topological weighting [77]. RRTs were performed for each single-gene alignment (note that all tRNA genes, due to their short length, were concatenated, and here considered like a single-gene alignment for analytic purposes), as well as for the mt and nuclear data sets at both the nucleotide and amino acid levels. In the case of protein-coding genes at the nucleotide level, tests were carried out (*i*) taking into account all codon positions and (*ii*) without first and third-codon positions of mt genes and third positions of nuclear genes. Genetic distances were estimated with K2P [78] and Poisson [75] models at the nucleotide and amino acid levels, respectively. We defined the three salamander species used in the timetree analysis as outgroup, and divided frogs into three assemblages: species-rich clades within Neobatrachia (Ranoides and Nobleobatrachia), species-poor neobatrachian lineages (*Heleophryne*, *Calyptocephalella*, *Lechriodus* and *Sooglossus*), and non-neobatrachian relatives (Amphicoela, Discoglossoidea, Pipoidea, and Pelobatoidea). RRTs were performed among these three assemblages, as well as between all neobatrachians versus non-neobatrachians.

#### Branch length comparisons

We optimized model parameters and branch lengths separately for the mt and nuclear data sets both at the nucleotide and amino acid levels using RA × ML v.7.0.4 [58], and constraining the topology to the preferred ML tree as recovered based on the combined nucleotide and amino acid data sets. In order to compare the branch-specific bias of mt versus nuclear branch lengths, the ratios between branch lengths (mt / nuclear) were calculated for each individual internal and terminal branch in the nucleotide- and amino acid-based trees, separately. The estimated ratios were subjected to one-way ANOVAs, after being log-transformed to meet the assumptions of normality and homogeneity of variance. The first ANOVAs compared neobatrachians against non-neobatrachians. For the second ANOVAs, neobatrachians were further divided into species-rich and species-poor lineages (following the scheme of RRT analyses; see above), and orthogonal contrasts were used to compare the three groups. All statistical analyses were performed with IBM SPSS Statistics, release 19.0.0.1.

#### Detecting changes in selective pressure

Simulation studies have shown that analyses of selection coefficients are rather robust to sequence divergence [79] (as is the case in the present study), having been successfully used in various studies with highly divergent species (*e.g.,*[80]). In order to understand whether acceleration of evolutionary rates in neobatrachians is due to changes in selective pressure, we tested alternative models with different assumptions about ratios of non-synonymous/ synonymous substitution rates (ω). The software PAML v.3.15 [81] was used to estimate the likelihood and the ω values of different models derived from the preferred topology (Figure 1) and sequence information from single-gene alignments with all codon positions, as well as the mt and nuclear nucleotide data sets. Branch lengths were first optimized for each data set assuming a single ω for the whole tree, and they were fixed when all other parameters were estimated under alternative models. The null model had a single ω value for all branches, and it was compared against four alternatives, which allowed a second ω value on (*i*) the stem branch of Neobatrachia, (*ii*) all neobatrachian branches, (*iii*) Ranoides, or (i*v*) Nobleobatrachia (including their stem branch)*.* Given that the alternative hypotheses nest the null model, a likelihood ratio test (LRT) was used to determine their significance [82]. To gain insight into the obtained results, we additionally allowed ω to vary on main non-neobatrachian lineages (including their stem branch): (*v*) Amphicoela, (*vi*) Discoglossoidea, (*vii*) Pipoidea, (*viii*) Pelobatoidea, or (*ix*) the stem branch of Pelobatoidea. These additional five models were compared against the null model by LRT, and, in addition, all 10 (non nested) models were compared simultaneously using the AIC [68].

#### Functional analysis of neobatrachian amino acid synapomorphies

Ancestral character states were reconstructed using MrBayes v3.1.2 [61] based on single-gene protein alignments, for (*i*) Neobatrachia, (*ii*) its closer sister group (Pelobatoidea), and (*iii*) their last common ancestor. The three hypothetical ancestral sequences were compared against each other to identify synapomorphic amino acid changes in Pelobatoidea and Neobatrachia, taking only into account the sites with reliably reconstructed states (we empirically found that p > 0.75 offered a good balance between the number of predictions and their corresponding reliability). A two-sided binomial test was used to assess the asymmetrical distribution of molecular synapomorphies between the two clades. In addition, to further understand if molecular synapomorphies of neobatrachians (or pelobatoideans) were associated to particular regions of the proteins, we predicted the accessibility to solvent and the occurrence at the different trans-membrane regions for each of the identified synapomorphic sites. Solvent accessibility was calculated through BLAST searches against the PDBFINDER2 database [83], and trans-membrane helices of genes were predicted with TMHMM v.2.0 [84].

#### Data availability

The newly determined mt (JF703228-34) and nuclear (JF703235-51) sequences are available at NCBI (http://www.ncbi.nlm.nih.gov/genbank/). The data sets used in this study (combined nucleotide, combined amino acid, mt nucleotide, mt amino acid, nuclear nucleotide, and nuclear amino acid data sets) as well as the .xml file used for the BEAST analyses can be accessed in the Dryad Digital Repository (doi:10.5061/dryad.3qd54).

## Results and discussion

### New mitochondrial genomes and nuclear data

We newly determined the complete nucleotide sequence of the light strand of the mt genome of one pelobatoid (*Pelodytes punctatus*; JF703231) and five neobatrachian species (*Helephryne regis,* JF703229; *Lechriodus melanopyga,* JF703230; *Calyptocephalella gayi,* JF703228; *Telmatobius bolivianus,* JF703234; *Sooglossus thomasseti,* JF703233), as well as the nearly complete mt genome of *Sooglossus sechellensis* (JF703232). We also determined partial sequences of several nuclear genes (see Additional file 1 for details) in order to construct a nuclear data matrix that complemented the mt sequence data set. Main structural features of the newly sequenced mt genomes are highlighted below, and other features can be found in Additional file 2.

The gene order of the mt genome in *Pelodytes punctatus* follows the consensus of vertebrates and other reported pelobatoids [27,47] (Figure 1). *Calyptocephalella gayi, Telmatobius bolivianus, Sooglossus thomasseti* and *S. sechellensis* conform to the consensus mt gene order of neobatrachians (Figure 1). The neobatrachian and vertebrate consensus mt gene orders differ in the relative position of the *trnL(CUN)*, *trnT* and *trnP* genes, which in neobatrachian mt genomes are found next to the *trnF* gene (forming the *LTPF* tRNA cluster) downstream the control region [85] (Figure 1). The mt genome of *Lechriodus melanopyga* follows the consensus neobatrachian gene order with the only exception of the location of the putative origin of replication of the light strand in a 218 bp-long intergenic spacer between the genes *trnY* and *cox1* (Figure 1). *Heleophryne regis* departs from the consensus order of neobatrachians in two regions. The *trnM* gene is pseudogenized (Figure 1; Ψ^M^) in its ancestral location (*IQM* tRNA gene cluster) because the anticodon has a deletion. The functional *trnM* appears within the *WANCY* tRNA gene cluster, which is rearranged as *ANCMWY*, without changes in the coding strands (Figure 1). The putative origin of the replication of the light strand (Figure 1; O_L_) is located in a 165 bp-long intergenic spacer between *trnW* and *trnY* genes (Figure 1). Interestingly, the two described new mt gene rearrangements are associated with origins of replication, which are considered hot spots for gene order change in the vertebrate mt genome [26,86,87].

These two newly reported gene rearrangements are consistent with the tandem duplication-random loss model [88,89], which is considered the main mechanism of gene order change in vertebrate mt genomes [26,87]. The tandem duplication-random loss model is reinforced by the presence of the *trnM* pseudogene, which remains in the ancestral location of *trnM* in *H. regis* (Figure 1). Other *trnM* pseudogenes have been reported in mt genomes of several members of the frog family Mantellidae [90,91], and several fishes including parrot fishes of the family Scaridae [92], *Carapus bermudensis* (Ophidiiformes; [93]), and *Diaphus splendidus* (Myctophiformes; [94]). It has been suggested that these pseudogenes are maintained because they are needed for the posttranscriptional processing of the *nad2* gene [91,92].

The mt gene order of *Heleophryne regis* is key for understanding the evolution of mt genome rearrangements in neobatrachians given that this species is basal in this clade (see reconstructed phylogeny in Figure 1). The mt gene order of *H. regis* has the characteristic *LTPF* tRNA gene cluster, which can, thus, be confidently considered a molecular synapomorphy of all neobatrachians. We speculate that the long intergenic spacer between *trnP* and *trnF* genes found in *H. regis* could be a remnant of the ancestral tandem duplication and random loss event by which the *trnL(CUN)*, *trnT* and *trnP* genes moved to the *LTPF* tRNA cluster in the origin of Neobatrachia [85].

### Phylogenetic relationships

Due to reported long-branch attraction effects in reconstructed anuran phylogenies based on mt sequence data [27], the concatenated data set of all mt and all nuclear single-gene alignments was trimmed to only retain most conserved positions (combined nucleotide data set: 11,136 positions). Based on the combined nucleotide data set with five partitions, both ML (-*ln*L=76,155.99) and BI (-*ln*L=76,547.00 for run 1; -*ln*L=76,548.29 for run 2) arrived at an identical topology (Figure 1), which is our best hypothesis for frog phylogenetic relationships. Phylogenetic analyses based on the combined amino acid data set (data not shown) also recovered the same topology (Figure 1). Five major clades of frogs were recovered with high support: non-neobatrachian lineages branched off successively as (*i*) Amphicoela (*Ascaphus* + *Leiopelma*), (*ii*) Discoglossoidea, (*iii*) Pipoidea and (*iv*) Pelobatoidea, which was the sister group of (*v*) Neobatrachia, in agreement with recent molecular studies [36,37,39,66]. Internal relationships within Discoglossoidea [28], Pipoidea [36,37,39,66], and Pelobatoidea [95] fully agreed with recent morphological and molecular analyses.

Neobatrachia has traditionally been acknowledged as monophyletic [96,97], a fact that has been corroborated by most morphological [34,35] and molecular [36,37,39] studies, including the present work (Figure 1). The reconstructed tree recovered *Heleophryne* as sister group to all other neobatrachians, as reported by most recent molecular studies [25,41,66], and this placement received moderate support from ML and maximal from BI (Figure 1). The Australasian *Lechriodus* and the South American *Calyptocephalella* were recovered together in the same clade, and both as sister group to Nobleobatrachia (Figure 1). The two Seychellois *Sooglossus* species were grouped together as sister group of Ranoides (as early suggested by Savage [98]) (Figure 1). Although support for this relationship was maximal for BI, it received only moderate support for ML. Notably, other recent molecular studies failed to resolve the relative position of sooglossids with confidence [25,39,41,66].

### Estimation of divergence times

**Figure 2:**
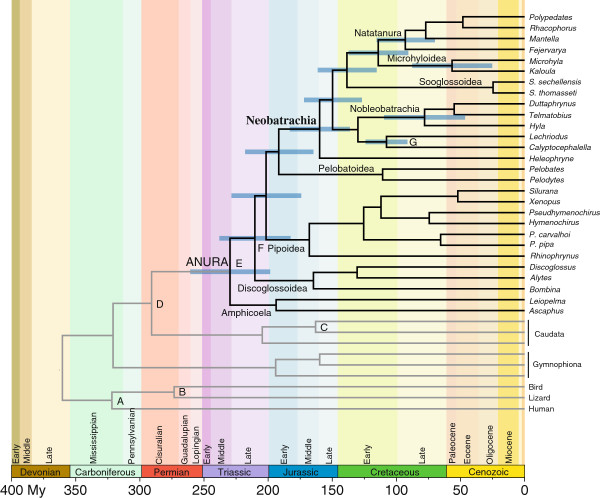
**Timetree with age estimates of major divergence events among frogs.** The tree was estimated using Bayesian relaxed dating methods (BEAST) and based on the combined nucleotide data set. Outgroup species are depicted with grey branches, horizontal blue bars represent 95% credibility intervals of relevant nodes, and calibration constraints are indicated on the corresponding nodes (A to G; see Additional file 2). Scale axis is in millions of years (My).

The two independent BEAST analyses gave very similar estimates of divergence times for each node (mean difference between runs was 0.638 ± 0.687 million years), and these estimates roughly agreed with other recent studies of divergence times among anurans [66,67,99]. The obtained estimates of divergence among amphibians were especially in agreement with those of a recent BEAST analysis [99], despite the differences in taxon sampling, choice of molecular markers, and calibration points. The origin of crown-group Anura was inferred in the Middle Triassic (about 230 mya) (Figure 2), and the initial diversification of non-neobatrachian frogs (successive branching of Amphicoela, Discoglossoidea, Pipoidea, and Pelobatoidea) in the Late Triassic – Early Jurassic (about 210–192 mya) (Figure 2), confirming other recent molecular dating studies [43,99]. The split between Neobatrachia and Pelobatoidea was dated in the Late Triassic – Early Jurassic, before the initial break-up of Pangaea (mean 192 mya; 95% CI 219–166), and the initial neobatrachian diversification that led to Ranoides and Nobleobatrachia in the Late Jurassic – Early Cretaceous (160–130 mya) (Figure 2) in agreement with divergence time estimates of other recent studies [33,67,99-101].

Our estimates were younger than those inferred by earlier studies [67,99,100] that used MultiDivtime [102,103], even though the 95% credibility intervals (CI) mostly overlapped. These discrepancies could be due to differences of both programs in methodological assumptions of rate change (auto-correlated in MultiDivtime, uncorrelated in BEAST), implementation of evolutionary models and prior calibrations, and techniques to calculate credibility intervals [104]. Moreover, molecular dating estimates are much older than the first neobatrachian fossils (dated in the Early Cretaceous; [105]), indicating either that currently available fossils might be a poor indicator of this particular branching event [105] or that the molecular dating could be overestimated [106].

### Congruence between mt- and nuclear-based phylogenies

**Figure 3:**
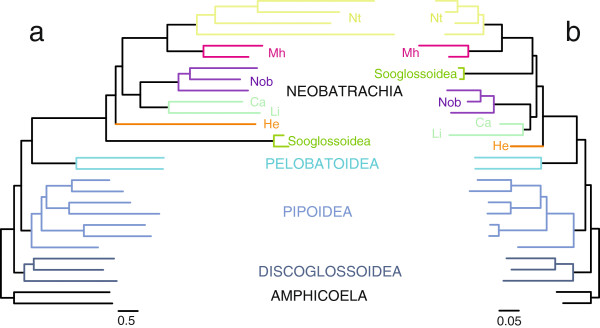
**ML phylograms derived from separate (a) mt and (b) nuclear nucleotide data sets.** Neobatrachian lineages are abbreviated as Ca, Calyptocephalellidae, He, Heleophrynidae; Li, Limnodynastidae; Nob, Nobleobatrachia; Mh, Microhyloidea; and Nt, Natatanura. Note that the most conspicuous differences between both trees are (*i*) the relative phylogenetic position of Sooglossoidea, (*ii*) the scale bar (substitutions/ site), which is proportionally 10 times larger in (**a**) than in (**b**), and (*iii*) that neobatrachian branches are distinctively longer in (**a**) than in (**b**).

Separate phylogenetic analyses of the mt (13,580 positions) and nuclear (7,083 positions) nucleotide data sets rendered two almost identical topologies (see Figure 3) with levels of support only slightly lower than those obtained in the phylogenetic analysis based on the combined nucleotide and amino acid data sets (not shown). The recovered phylogenetic tree based on the mt nucleotide data set (Figure 3a) placed sooglossids as the most basal neobatrachians (branching off before *Heleophryne*), which is likely an artifact due to the attraction of the extremely long branch leading to sooglossids towards that of *Heleophryne* and the stem branch of Neobatrachia (which is the longest branch in the tree) [23]. Additionally, this phylogenetic tree (Figure 3a) favored with low statistical support alternative relationships for (*Duttaphrynus* + (*Telmatobius* + *Hyla*)) within Nobleobatrachia. Interestingly, the phylogenetic analysis of the mt amino acid data set differed from our best hypothesis (Figure 1) only in the basal position of sooglossids within Neobatrachia (not shown). The phylogenies reconstructed based on the nuclear nucleotide (Figure 3b) and nuclear amino acid (not shown) data sets recovered our best hypothesis for frog phylogenetic relationships (Figure 1).

In addition to assessing topological congruence, the separate analyses of the mt and nuclear data sets (both at nucleotide and amino acid levels) offer further information on the mode of evolution of these two different genetic systems. The comparison of mt- and nuclear-based trees (Figure 3) reveals that branch lengths in the mt tree (Figure 3a), which ultimately correspond to the underlying substitution rates, are on average 3.29 (0.42 – 12.15) and 2.82 (0.31-18.19) times longer than those in the nuclear tree (Figure 3b) at the nucleotide and amino acid levels, respectively. This confirms previous evidence of higher substitution rates in the mt DNA of metazoans [10,12,13]. More importantly, neobatrachians exhibit much longer branches in the mt trees compared to their non-neobatrachian relatives, with *Heleophryne*, *Sooglossus*, and natatanuran species having the longest branches. In contrast, neobatrachians do not display such conspicuous long branches in the nuclear tree, and a lineage-specific pattern of branch lengths is only subtle (Figure 3b).

### Relative-rate tests and comparison of branch lengths

In order to test whether the mt substitution rate is significantly higher in neobatrachians, and to study putative lineage-specific rate changes in nuclear genes, we conducted RRTs, as well as a direct comparison of the ratios of the lengths of the same branch in the mt- and nuclear-based phylogenies (both at the nucleotide and amino acid levels). Overall, RRTs clearly showed that neobatrachians had significantly higher mt substitution rates compared to non-neobatrachians (K2 versus K1; Table 1). RRTs were applied to 16 individual mt gene alignments (13 protein-coding genes, 2 rRNA genes, and a single alignment combining all tRNA genes) at the nucleotide level, and neobatrachians had higher mean relative rates in all cases but the *nad3* gene (Table 1). Differences were significant (p < 0.05) for 12 out of 16 mt genes, and highly significant (p < 0.001) for the concatenation of all mt genes (mt nucleotide data set; Table 1). RRTs of individual nuclear genes at the nucleotide level showed that six out of nine genes had higher mean substitution rates for neobatrachians (although significant only in the case of the genes *rag2* and *slc8a1*). Genes *h3a*, *pomc*, and *rho* showed lower rates for Neobatrachia, but differences were non-significant (Table 1). Notably, the concatenation of all nuclear genes (nuclear nucleotide data set; Table 1) gave significantly higher rates for neobatrachians than for non-neobatrachians.

**Table 1:** Results from relative-rate tests based on nucleotide single-gene alignments and mt and nuclear nucleotide data sets

	**Mean weighted substitution rates**				**Probability of relative-rate tests**			
**gene**	**K1**	**K2**	**K3**	**K4**	**p (1 vs. 2) p < 0.05**	**p (1 vs. 3) p < 0.0167**	**p (1 vs. 4) p < 0.0167**	**p (3 vs. 4) p < 0.0167**
*atp6*	0.427863	0.473377	0.481832	0.455958	0.014730*	0.005722*	0.105996	0.139169
*atp8*	0.548420	0.597954	0.603741	0.598698	0.262944	0.238598	0.273487	0.916317
*cob*	0.357846	0.395422	0.400600	0.376129	0.153630	0.127295	0.461317	0.438624
*cox1*	0.258230	0.291037	0.294927	0.288984	***1.26·10***^-4^*	***4.86·10***^-5^*	***4.59·10***^-5^*	0.475097
*cox2*	0.279140	0.349751	0.355794	0.328478	***6.31·10***^-6^*	***3.76·10***^-6^*	***5.41·10***^-4^*	0.068572
*cox3*	0.289143	0.322240	0.328289	0.304404	0.007560*	0.002549*	0.170152	0.046288
*nad1*	0.371027	0.401329	0.413424	0.383640	0.023124*	0.002486*	0.296321	0.020798
*nad2*	0.507982	0.547513	0.548407	0.525736	0.025438*	0.026856	0.251499	0.170942
*nad3*	0.434548	0.430927	0.434119	0.433411	0.869559	0.985402	0.956541	0.974496
*nad4*	0.431683	0.557581	0.560891	0.590991	***1.00·10***^-7^*	***1.00·10***^-7^*	***1.00·10***^-7^*	0.050825
*nad4L*	0.497599	0.631415	0.643682	0.588595	***8.27·10***^-4^*	***6.30·10***^-4^*	0.009870*	0.150316
*nad5*	0.411432	0.519950	0.517924	0.538763	***1.00·10***^-7^*	***1.00·10***^-7^*	***1.00·10***^-7^*	0.081618
*nad6*	0.463362	0.505592	0.512071	0.518343	0.088626	0.056897	0.026638	0.765287
*rrnS*	0.191816	0.26154	0.265755	0.253359	***1.00·10***^-7^*	***1.00·10***^-7^*	**9,73*·10***^-3^*	0.267807
*rrnL*	0.193460	0.217439	0.219738	0.214547	0.005620*	0.003273*	0.0176213	0.516844
tRNAs	0.272203	0.309978	0.312829	0.299828	0.001434*	0.000809*	0.010368*	0.221773
**all mt genes**	0.330116	0.379173	0.384017	0.372441	***1.00·10***^-7^*	***1.00·10***^-7^*	***1.00·10***^-7^*	0.001898*
*bdnf*	0.162298	0.183220	0.183143	0.188813	0.063971	0.066639	0.019606	0.425053
*cxcr4*	0.305898	0.310540	0.310616	0.317277	0.749761	0.760622	0.463425	0.635922
*h3a*	0.072185	0.071813	0.074309	0.069597	0.967280	0.831715	0.787527	0.662899
*pomc*	0.383197	0.374114	0.370759	0.381447	0.747452	0.671607	0.956266	0.667038
*rag1*	0.278115	0.298226	0.297262	0.300048	0.177027	0.210499	0.172426	0.837948
*rag2*	0.436022	0.493728	0.494946	0.512607	0.002539*	0.003285*	***2.38·10***^-4^*	0.314455
*rho*	0.177431	0.164405	0.167539	0.177528	0.444978	0.596616	0.995982	0.609647
*slc8a1*	0.236296	0.253733	0.253592	0.255299	0.029208*	0.041528	0.030535	0.840326
*slc8a3*	0.223439	0.233366	0.232416	0.238298	0.280968	0.356872	0.115003	0.460431
**all nuc genes**	0.067158	0.075283	0.074880	0.072843	0.036588*	0.055309	0.164760	0.607197

K values are mean weighted substitution rates for (1) non-neobatrachians, (2) all neobatrachians, (3), species-poor neobatrachians (Heleophrynidae, Calyptocephalellidae, Limnodynastidae, Sooglossoidea), and (4) species-rich neobatrachians (Ranoides and Nobleobatrachia). Probabilities (p) of relative-rate tests correspond to the comparisons between the different groups (shown in parentheses). Statistically significant results (p < 0.05; or p < 0.05/3 = 0.0167 to correct for multiple testing) are in bold italics and marked with an asterisk. Abbreviations of mt genes follow [44]; see main text for full names of nuclear genes.

Additional RRTs based on the same nucleotide data sets mentioned above were performed to discriminate among three alternative hypotheses regarding the origin of the higher mt rates of neobatrachians: (*i*) mt substitution rates became accelerated in the origin of the clade and were maintained higher ever since; (*ii*) the higher substitution rates are specific to species-poor neobatrachian lineages; and *(iii)* the higher substitution rates are specific to species-rich neobatrachian lineages. In the second and third hypotheses, rate acceleration would be either punctual at the base of Neobatrachia (and thus, not present in species-rich lineages), or it would be a specific feature of the species-rich Ranoides and Nobleobatrachia (as suggested by [25]), respectively. The comparison of species-rich (Ranoides, Nobleobatrachia) and species-poor (*Heleophryne*, *Calyptocephallela, Lechriodus, Sooglossus*) neobatrachian groups against non-neobatrachian relatives (K4 versus K1, and K3 versus K1, respectively; Table 1) showed that both groups had consistently higher mt substitution rates, which were significant after Bonferroni correction for multiple comparisons (p < 0.05/ 3 = 0.0167). No significant differences in mt substitution rates were observed between species-rich and species-poor neobatrachians (K3 versus K4; Table 1). However, for the concatenation of all mt genes (mt nucleotide data set), species-poor neobatrachian lineages showed overall significantly higher rates compared to species-rich neobatrachians. In the case of the different nuclear genes, the separate comparison of species-rich and species-poor neobatrachians against non-neobatrachians gave similar results: the *rag2* gene had consistently higher mean rates for both neobatrachian groups, but the higher neobatrachian rates found for the *slc8a1* gene failed to be significant due to the lower significance threshold. No differences in nuclear substitution rates were observed among neobatrachians (K3 versus K4; Table 1). The concatenation of all nuclear genes (nuclear nucleotide data set) rendered non-significant comparisons (Table 1). All previously mentioned RRTs were also performed with protein-coding genes translated into amino acids, and produced consis-tent results with those obtained based on nucleotides (see Additional file 3).

In order to disentangle the contribution of the different codon positions to evolutionary rate acceleration in neobratrachians, we performed additional RRTs based on the mt (lacking first and third codon positions) and nuclear (lacking third codon positions) portions of the combined nucleotide data set, respectively (see Additional file 3). As expected, exclusion of most variable positions from the analyses produced lower mean weighted substitution rates in both mt and nuclear genes. This would in part explain the better performance of the combined nucleotide data set in resolving anuran phylogeny. Nevertheless, RRT results showed that conserved codon positions also contribute significantly to neobatrachian-specific rate acceleration, displaying similar trends to those experienced by more variable codon positions (see Additional file 3).

In order to compare the branch-specific rate bias of mt versus nuclear genes and quantify the acceleration of mt rates in Neobatrachia, we calculated the ratio of the lengths of each branch in the mt- and nuclear-based trees (both at the nucleotide and amino acid levels). For the ANOVAs, the ratios were log-transformed to meet the assumptions of normality (Shapiro-Wilk’s test on residuals p > 0.05) and homogeneity of variance (Levene’s test p > 0.05). A first set of ANOVAs supported a significant difference of the ratios of all neobatrachian versus all non-neobatrachian branches (p = 0.012 and 0.001 for nucleotide- and amino acid-based trees, respectively). The higher mt / nuclear ratios of neobatrachians (mean ± standard deviation for nucleotide- and amino acid-based trees, respectively: 3.90 ± 2.38 and 3.90 ± 3.67) compared to those of non-neobatrachians (2.66 ± 2.66 and 1.67 ± 1.27), showed that relative to the nuclear genome, the mt genome is approximately 46 and 130% more accelerated in Neobatrachia at the nucleotide and amino acid levels, respectively. The striking difference in percentage estimates between nucleotide and amino acid levels could be explained by saturation of more variable codon positions in the former. A second set of ANOVAs found significant differences in branch length ratios between species-poor neobatrachians, species-rich neobatrachians, and non-neobatrachians (p = 0.039 and 0.002 for nucleotide and amino acids, respectively). Further orthogonal contrasts found significant differences when non-neobatrachians were compared against species-poor (p = 0.027 and 0.001; nucleotide and amino acid levels, respectively) and species-rich (p = 0.049 and 0.011) neobatrachians. However, no significant differences were found between neobatrachian groups (p = 0.650 and 0.291).

Estimation of branch length ratios based on the mt (lacking first and third codon positions) and nuclear (lacking third codon positions) portions of the combined nucleotide data set rendered the same patterns of rate heterogeneity as derived from comparative analyses based on all codon positions and amino acids. Interestingly, however, when considering only conserved codon positions, we inferred that the mt genome is approximately 88% more accelerated in Neobatrachia relative to the nuclear genome. This percentage is closer to that obtained based on amino acid data, and further supports that the one based on all positions may be underestimated due to saturation.

Overall, RRTs support a statistically significant acceleration of the mt substitution rate at the origin of neobatrachians, which is shared by both species-poor and species-rich lineages. This result not only corroborates previous studies that suggested an unequal distribution of mt substitution rates between non-neobatrachian and neobatrachian frogs [25-28,33], but also characterizes the evolutionary dynamics of the shift in rates within neobatrachians. The inferred evolutionary pattern is further corroborated by ANOVA results indicating that neobatrachian mt lineages are evolving 88 to 130% faster than non-neobatrachian mt lineages with respect to nuclear rates. Nuclear genes also exhibited an overall significant substitution rate acceleration, although much lower in absolute terms than the one experienced by the mt genome. In fact, the nuclear pattern was not as evident as that of the mt genes, since neobatrachian-specific higher substitution rates were found to be significant in only two out of nine nuclear genes. This might be the result of the different properties of mt and nuclear genomes, such as the recombination rate (very limited in mt DNA; [18]) or the different effective population size (smaller in mt DNA; [18]), which can influence the effectiveness of selection upon these two genetic systems [107]. An alternative explanation is that although substitution rate acceleration is general for both mt and nuclear genomes, our results are biased by the use of particular nuclear genes, which cannot represent the complexity of the entire nuclear genome, with genes obviously subjected to very disparate selective regimes [108].

### Changes in selective pressure and molecular synapomorphies

The assumption of a single selective coefficient (ω) for the frog tree (Figure 1) (null model) rendered ω values well below 1 for all different mt and nuclear genes (0.005-0.16) (Table 2), indicating the action of purifying selection to maintain gene function [14]. To understand whether observed acceleration of the mt substitution rate in neobatrachians could be due to changes in selective pressure, and to compare the strength of selection acting on the mt and nuclear genomes, we tested for putative changes in the selective regime under four different scenarios for the Neobatrachia. All outcomes are available in the Additional file 3, and main results are here highlighted.

**Table 2:** Results from branch models that assume branch-specific changes in the selection coefficient (ω) in Neobatrachia, based on nucleotide single-gene alignments and mt and nuclear nucleotide data sets

**models**	**null model**	**"Neob-stem"**		**"All Neob"**		**"All Nobleob"**		**"All Ranoides"**	
**gene**	**Backgr**	**Backgr**	**Branch**	**Backgr**	**Branch**	**Backgr**	**Branch**	**Backgr**	**Branch**
*atp6*	0.03086	0.03076	0.03585	0.03045	0.03126	0.03086	1.1225	0.03082	0.04203
*atp8*	0.1481	0.14592	1.50841	0.13361	0.16694	0.14838	0.13614	0.14683	0.35429
*cob*	0.03357	***0.03313***	***0.06875***	0.03302	0.0342	0.03347	0.04419	0.03353	0.0431
*cox1*	0.01129	***0.011***	***0.03737***	***0.00783***	***0.0144***	0.01122	0.02381	***0.01115***	***0.03898***
*cox2*	0.02236	0.02224	0.02642	0.02186	0.02271	0.02234	0.0236	0.02227	0.03366
*cox3*	0.02741	***0.02704***	***0.08793***	***0.02482***	***0.03046***	0.02727	0.03895	0.02733	0.05843
*nad1*	0.03005	***0.02977***	***0.05077***	***0.02823***	***0.03211***	0.03001	0.03416	0.02996	0.04253
*nad2*	0.02817	0.02813	0.03252	0.02803	0.02835	0.02818	0.02814	0.02814	0.03007
*nad3*	0.06499	0.06457	0.14425	0.06435	0.06582	0.06467	0.10545	0.06499	0.93185
*nad4*	0.03632	0.03622	0.0392	0.0358	0.03675	0.03626	0.04061	0.0363	0.03703
*nad4L*	0.04471	0.04458	0.04792	0.04662	0.04311	0.04474	0.04294	0.04468	0.04637
*nad5*	0.03516	0.03503	0.03793	0.03459	0.03571	0.0351	0.03749	0.03511	0.03836
*nad6*	0.02901	0.02895	0.03107	0.02753	0.03081	0.02897	0.03286	0.02895	0.03359
all mt genes	0.04693	***0.04632***	***0.08313***	***0.04483***	***0.04914***	***0.04673***	***0.06435***	***0.04667***	***0.07065***
*bdnf*	0.04595	0.04723	0.01186	0.04393	0.04979	0.04577	0.05385	0.04545	0.11214
*cxcr4*	0.06461	0.06454	0.06745	0.06028	0.07344	0.06588	0.03076	0.06441	0.0748
*h3a*	0.00476	0.00483	0.0001	0.00599	0.00283	0.005	0.0001	0.00488	0.0001
*pomc*	0.08214	0.0808	0.23558	0.09098	0.07043	0.08298	0.05716	***0.08548***	***0.02649***
*rag1*	0.05736	0.05778	0.03692	***0.05249***	***0.06532***	0.05772	0.0438	***0.05802***	***0.01704***
*rag2*	0.16003	0.15849	0.24864	0.16786	0.1515	0.16003	0.0001	0.1612	0.04584
*rho*	0.0903	0.092	0.02424	***0.07483***	***0.11389***	0.09175	0.02681	0.09226	0.04981
*slc8a1*	0.03996	***0.04067***	***0.0001***	***0.03588***	***0.04702***	0.0402	0.03164	0.04055	0.01522
*slc8a3*	0.02649	0.02684	0.0144	0.02623	0.02707	0.02664	0.01996	0.0268	0.01046
all nuc genes	0.06908	0.06922	0.06195	***0.06426***	***0.07719***	0.06976	0.04153	***0.06984***	***0.03298***

The table shows ω values estimated for the whole frog tree (null model) and values estimated for specific (Branch) and remaining background (Backgr.) branches under the four alternative models tested. Bold italics highlight results that are significantly different to the null model (LRT p < 0.05). See Additional file 3 for full details.

For all mt genes, the independent ω values inferred for the stem branch of Neobatrachia were always higher than those estimated when a single ω was assumed for the frog tree (null model) (Table 2). These differences were only significant (LRT p < 0.05) for the genes *cob, cox1, cox3,* and *nad1*, as well as for the combination of all mt genes (Table 2). The independent ω estimates of mt genes under alternative models for “all Neobatrachia”, “all Ranoides” and “all Nobleobatrachia” branches were generally higher than those of the null model, but unlike the model of the stem branch Neobatrachia, ω was not higher for every mt gene, and fewer individual genes gave significant results (3, 1, and none for the three alternative models, respectively; see Table 2). These outcomes suggest that purifying selection acting on mt proteins could have been relaxed in neobatrachians.

In order to understand the relative support of the first four models tested (within Neobatrachia), and to further investigate the relevance of the obtained results, we compared all 10 models using the AIC. For the combination of all mt genes, the model of relaxed selection in the stem branch of Neobatrachia was clearly better than the remaining models (Table 3). All other models showed a difference of AIC values (ΔAIC) higher than 10: ΔAIC=48 for the second best model (independent ω for Pipoidea), ΔAIC=56 for the third (independent ω shared by all neobatrachian branches), ΔAIC=61 for the fourth (independent ω for Ranoides), etc. (Table 3). A notable exception to the above pattern was the gene *cox1;* despite evidence of relaxed selection in the stem branch of Neobatrachia, the comparison of all 10 models for *cox1* strongly favored the relaxation along all branches of Neobatrachia (ΔAIC to the rest of models was always >10, and up to 44; Table 3).

**Table 3:** Comparison of all 10 branch models that assume branch-specific changes in the selection coefficient (ω) (including the null model), based on nucleotide single-gene alignments and the mt and nuclear nucleotide data sets

	**null model**	**“Neob-stem”**	**“All Neob”**	**“All Ranoides”**	**“All Nobleob”**	**“All Pipoidea”**	**“Pelob-stem”**	**“All Pelob”**	**“ All Discogl”**	**“All Amphic”**
**genes**										
*atp6*	0	2	2	2	2	1	2	2	2	2
*atp8*	5	4	4	6	7	0	7	7	4	5
*cob*	7	0	9	9	9	7	9	9	3	9
*cox1*	42	27	0	36	43	11	44	43	27	44
*cox2*	6	7	8	7	8	8	8	6	0	8
*cox3*	5	0	2	7	7	1	5	7	5	7
*nad1*	3	1	1	4	5	0	5	5	5	5
*nad2*	0	2	2	2	2	2	2	2	2	2
*nad3*	0	0	2	2	1	2	2	0	2	1
*nad4*	0	2	2	2	2	1	2	2	2	2
*nad4L*	0	2	1	2	2	1	2	2	2	1
*nad5*	0	1	1	2	2	2	2	1	2	2
*nad6*	0	2	0	2	2	1	2	2	2	2
all mt genes	78	0	56	61	68	48	77	80	66	79
*bdnf*	1	0	2	2	3	3	3	2	2	1
*cxcr4*	10	12	10	12	9	0	4	9	4	11
*h3a*	1	2	1	2	1	0	2	3	2	2
*pomc*	7	5	5	0	8	8	8	8	7	5
*rag1*	8	8	3	4	9	6	4	0	8	5
*rag2*	7	7	7	7	9	8	8	7	8	0
*Rho*	3	3	0	3	3	4	4	2	3	1
*slc8a1*	15	10	12	13	16	14	17	16	15	0
*slc8a3*	15	16	17	15	16	16	16	14	16	0
all nuc genes	32	33	14	18	24	34	28	19	34	0

The table shows the differences in AIC values among all 10 models.

For nuclear genes, most of the estimated independent ω values in all of the nine alternative models were lower than those of the null model, showing evidence of stronger purifying selection, although genes did not display a concordant pattern neither under particular models nor within specific genes (Table 2). However, there were two exceptions: (*i*) under the “all Neobatrachia” alternative model, relaxation of purifying selection on nuclear genes was frequently recovered (in six out of nine genes, even though it was statistically significant only for the genes *rag1, rho, slc8a1*, and the combination of all nuclear genes; LRT p<0.05); (*ii*) under the model of an independent ω for Amphicoela, for which relaxation of selection was also frequent (all nuclear genes except *h3a*, although the differences were only statistically significant for the genes *pomc, slc8a1, slc8a3*, and the combination of all nuclear genes) (Table 2). Using the concatenation of all nuclear genes, the comparison of the models assuming a second independent ω for (*i*) all Neobatrachia and (*ii*) Amphicoela favored the latter (ΔAIC=14; Table 3).

Overall, our results evidence the relaxation of purifying selection acting on mt DNA in Neobatrachia. Among all tested models, the assumption of relaxation at the stem branch leading to Neobatrachia outperformed the rest, although the very similar results obtained under the model of a general relaxation along the entire Neobatrachia indicates that this alternative hypothesis cannot be confidently rejected. In any case, these results suggest that overall relaxation in selection pressure could be, at least in part, responsible for the general acceleration of mt substitution rates at the origin of Neobatrachia, as found by RRTs and topological measures. Interpreting the results from nuclear genes was more complex because inferred relaxed selection along all neobatrachian branches could only explain the higher substitution rates found by RRTs for the gene *slc8a1*. Moreover, it is important to note that results derived from the comparison of different selection regimes should be taken with caution, because analyzed sequences are highly divergent and silent substitutions might be saturated, thus compromising the correct estimation of ω values [79].

Most identified amino acid synapomorphies corresponded to Neobatrachia in mt proteins (102 versus 49), whereas distribution of synapomorphies in nuclear proteins was only slightly higher in neobatrachians (24 versus 22). These differences were only significant (binomial test’s p < 0.05) for genes *cox1*, *nad5* and *rag2* (see Additional file 3)*.* This is in agreement with the results of the RRTs, which revealed overall higher substitution rates in neobatrachians, and a more pronounced acceleration in mt genes. Most mt synapomorphies corresponded to leucine, serine, and alanine in both Neobatrachia and Pelobatoidea (18, 13, and 11 for Neobatrachia; and 10, 10, and 7 for Pelobatoidea, respectively). In the nuclear genes, the most frequent synapomorphies for Neobatrachia were serine (4), glutamic acid (3), and lysine (3), whereas for Pelobatoidea, they were leucine (6) and aspartic acid (3) (see Additional file 3). To further understand how proteins of neobatrachians could have accommoda-ted the corresponding mutations, we investigated whether synapomorphic amino acids showed any particular pattern of exposition to solvent, or whether they were associated with specific domains of trans-membrane proteins. However, distribution of neobatrachian synapomorphic changes were not related apparently to these functional traits, with mutations being distributed in a more or less uniform manner along mt proteins.

### Could substitution rates be associated with life history traits, rates of diversification or mt gene rearrangements in frogs?

Our analyses recovered a fully resolved and robust phylogeny of frogs after new data on key lineages of Neobatrachia were added. These new lineages were essential to understand the origin of the observed higher substitution rates in neobatrachians. RRTs and branch length measures demonstrate the presence of a significant acceleration in both mt and nuclear substitution rates in Neobatrachia, which is shared by both species-rich and species-poor neobatrachian lineages.

The ultimate causes for among-lineage evolutionary rate variation are, in general, rather elusive. Several lines of evidence suggest that particular life-history traits may be responsible for rate variation [2], and at least, three main hypotheses have been put forward. (*i*) According to the generation time hypothesis, species with shorter generation times are expected to have higher substitution rates because their genomes are copied more often per time unit [109]. However, estimating generation time is often difficult, and thus, the age at sexual maturity and the time to first reproduction are used as proxies [109,110]. In frogs, generation time is generally short, and small- to medium-sized frogs typically reach sexual maturity in their first or second year of life [111]. Sexual maturity one year after egg-laying is common in many anuran species, including both neobatrachian and non-neobatrachian frogs (*e.g.*, *Bombina*, *Discoglossus*, *Hymenochirus* or *Xenopus*). On the other hand, several neobatrachians have longer generation times, such as *Heleophryne* (larval period of over 2 years; [40]), or *Anaxyrus canorus* (Bufonidae), whose females reach sexual maturity after 4–6 years [112]. Data on sexual maturity available from the AnAge database (build 12; [113]) does not support the existence of significant differences in age of sexual maturity between neobatrachian and non-neobatrachian frogs, either for males or females (Student’s t p = 0.530 and p = 0.754, respectively). However, these results should be taken with caution, as the available number of age estimates of sexual maturity data in AnAge is still insufficient to draw robust conclusions (N between 32–34 and 2–3 for neobatrachians and non-neobatrachian males and females, respectively).

(*ii*) The longevity hypothesis proposes that long-lived or late reproducing species will have lower rates of molecular evolution [114], as they are expected to have more effective DNA repairing mechanisms [115]. In most cases, maximum longevity data is derived from captive specimens, which have higher life expectancy that in the wild [116]. Available data from the AnAge database suggests that short- and long-lived species are present both among neobatrachian and non-neobatrachian frogs. The comparison of longevity between neobatrachians and non-neobatrachians did not render significant differences (Student’s t p = 0.066; N = 10 and 75 for neobatrachians and non-neobatrachians, respectively). Inferences derived from this incomplete data set have to be taken with caution, though.

(*iii*) The metabolic rate hypothesis holds that rates of evolution are correlated with the production of free radicals during respiration [117]. Metabolic rate is correlated with substitution rates in the frog family Dendrobatidae [4], but comparable data for other anuran groups is currently unavailable. A suitable proxy for metabolic rate might be genome size, which has often been shown to be inversely proportional to metabolic rate [118-120]. However, the comparison of genome sizes among anurans (data from the Animal genome size database; [121]) did not provide indications for consistently larger genome sizes in non-neobatrachians compared to neobatrachian frogs (Student’s t p = 0.770): C-values (mean ± standard deviation, minimum-maximum in parentheses) were 5.10 ± 3.65 pg (1.29-11.38 pg; N=9) and. 5.43 ± 2.59 pg (1.40-13.40 pg; N=27), respectively.

Some studies found a correlation of high substitution rates with more events of gene order rearrangements in the mt genome of some metazoans [122,123]. However, frogs do not conform to this pattern, because the mt substitution rate became accelerated in the origin of Neobatrachia, and this acceleration is not exclusive of Natatanura, where most mt gene rearrangements are found in frogs [90,124-127]. Furthermore, Kurabayashi et al. [90] found evidence for the absence of correlation between mt rates and number of gene rearrangements in one intensively studied lineage of neobatrachians (mantellid frogs from Madagascar).

Many other studies have found substitution rates to be correlated with species diversification [5-7,128], and three main hypotheses have been proposed to explain this correlation [6]. (*i*) Speciation is often associated with processes that can potentially increase substitution rates, such as adaptation to new environments or transient reductions in population sizes that reduce the efficiency of purifying natural selection [129,130]. (*ii*) Higher substitution rates could produce higher net diversification, both by increasing speciation rate and/ or by reducing extinction rate [6]. (*iii*) A third hypothesis rejects a causal relationship between substitution and diversification rates, and holds that this correlation is due to other factors that influence both rates simultaneously [131].

In frogs, it has been suggested that observed higher mt substitution rates of neobatrachians could be the product of faster recent speciation events in this clade (including more bottleneck events) [25]. In addition, Dubois [132] hypothesized that direct-developing species (mostly within Neobatrachia) would tend to have higher substitution rates, and this would in turn promote speciation. Alternatively, it has also been suggested that higher substitution rates of neobatrachians could be responsible for higher diversification rates, due to shorter generation times and/or higher metabolic rates [25]. Both species-rich and species-poor lineages of neobatrachians share higher mt substitution rates compared to non-neobatrachian relatives, but species diversity is highly unequally distributed among them, with most of the diversity corresponding to Ranoides and Nobleobatrachia [38]. Moreover, neobatrachians do not fit the hypothesis of the different reproductive modes [132] because, most direct-developers belong to species-rich clades, whereas species-poor neobatrachian lineages are mostly indirect-developers [34,132], but both groups share the presence of higher mt substitution rates. Therefore, the relationship between higher mt substitution and diversification rates in frogs remains elusive, and unless a rampant extinction of neobatrachian lineages external to currently species-rich clades (Ranoides and Nobleobatrachia) could account for the observed huge differences in diversity, it can be considered that substitution and diversification rates are decoupled in frogs. Unfortunately, the paucity of the current fossil record may hinder the answer to this question [105,133,134].

## Conclusions

Using both complete mt genomes and partial sequences of nine nuclear loci, we inferred a robust phylogeny of frogs. RRTs and branch length measures found compelling evidence of higher substitution rates in the mt genome of neobatrachian frogs, and a subtle (but significant) trend in nuclear genes. Phylogenetic analyses suggest that the origin of this rate acceleration began at the stem branch leading to Neobatrachia, in the Early-Middle Jurassic period. Because substitution rates are determined, to a great extent, by the balance between selection and genetic drift [8], we studied the changes in selective pressure in frogs, and found that purifying selection acting on most mt and some nuclear proteins might have been relaxed in Neobatrachia. Therefore, we suggest that this relaxation of purifying selection could explain, at least in part, the general rate acceleration observed in this group.

We do not exclude the possibility that our results are slightly affected by some sort of phylogenetic artifact [24], but the neobatrachian-specific higher mt substitution rates are reinforced by compelling evidence of relaxed purifying selection on mt proteins. Furthermore, our results show that selection might have been relaxed also in nuclear genes, and thus justify the higher substitution rates found in the genes *rag2* and *slc8a1* in neobatrachians. Data from additional nuclear genes, which are likely to be gathered soon in the context of genome sequencing initiatives, hold the key to confirm or reject a putative general acceleration of evolutionary rates in neobatrachian frogs. Likewise, the clarification of the causes that relaxed purifying selection would need further, in-depth studies that investigate intrinsic and extrinsic factors that might have modified the fitness landscape of gene function [135].

With the exception of few particular linages (*e.g.*, [4]), available data on life history traits for frogs is generally scarce and not representative for the main lineages within Anura. Available data suggests that no clear differences exist between neobatrachian and non-neobatrachian frogs with respect to generation time, longevity, or metabolic rate, but more data would be necessary to reliably test if substitution rates could be correlated with particular life history traits in frogs.

## Competing interests

The authors declare that they have no competing interests.

## Authors’ contributions

II carried out molecular lab work. II, DSM, and FA analyzed the data. II, DSM, FA, AO, MV, and RZ wrote the paper. All authors read and approved the final manuscript.

## Supplementary Material

Additional file 1 Taxa and GenBank accession numbers. Click here for file

Additional file 2 Supplementary information. Click here for file

Additional file 3 Additional results from RRTs, changes in selection, and molecular synapomorphies. Click here for file
