# Supplementary material for: The origin of modern frogs (Neobatrachia) was accompanied by acceleration in mitochondrial and nuclear substitution rates
Source: BMC Genomics. 2012 Nov 15;13:626. doi: 10.1186/1471-2164-13-626 (PMC3551647; doi:10.1186/1471-2164-13-626)
Supplement: Additional file 2 — Supplementary information. [file 1471-2164-13-626-S2.pdf]

Additional file 2: supplementary information for the manuscript "The origin of modern frogs (Neobatrachia) was accompanied by acceleration in mitochondrial and nuclear substitution rates"

Iker Irisarri, Diego San Mauro, Federico Abascal, Annemarie Ohler, Miguel Vences and Rafael Zardoya

1. Calibration points used for the estimation of divergence times

The following calibration points and distribution settings were used as priors:

A) Sauropsida-Synapsida split: Offset=312.3 million years ago (mya) as the minimum age for *Hylonomus* [1); log mean=2.2; log SD=0.424.

B) Archosauromorpha-Lepidosauromorpha split: Offset=259.7 mya as the minimum age for *Protorosaurus* [1]; log mean=2.4468; log SD=0.756.

C) Cryptobranchidae-Hynobiidae split: Offset=145.5 mya. Following Roelants et al. [2], we used a more conservative age estimate of *Chunerpeton* [3]; log mean=3.5; log SD=1.014. The soft maximum is 321 mya, corresponding to the upper limit for the Anura-Caudata split in San Mauro [4]. This is a more conservative upper bound than that suggested by Marjanović and Laurin [5].

D) Anura-Caudata split: Offset=249, which is the minimum age for the salientian *Triadobatrachus*, [6]; log mean=3.7; log SD=0.351; the soft maximum is 321 mya (see C).

E) Branching of Discoglossoidea: Offset=161.2 mya as the Middle-Late Jurassic boundary [7] corresponding to *Eodiscoglossus*, the first known Discoglossoidea [8]; log mean=3.6; log SD=0.532. The upper 95% IC value (soft maximum) is 249 mya (*Triadobatrachus*).

F) Branching of Pipoidea: Offset=145.5 mya as the minimum age for *Rhadinosteus*, the first-known pipoid [9, 10]; log mean=3.45; log SD=0.668. Soft maximum=249 mya (*Triadobatrachus*).

G) *Calyptocephalella-Lechriodus* split: Offset=52.8 mya as the oldest-known fossil of *Calyptocephalella* [10, 11]; log mean=4.2; log SD=0.2. The age of *Rhadinosteus* (145.5 mya; see F) is used as a conservative soft maximum.

2. New mitochondrial genomes

Like in most metazoans, all newly sequenced frog mt genomes encoded for 2 rRNAs, 22 tRNAs and 13 protein-coding genes [12, 13]. All tRNA genes could be folded into the typical cloverleaf secondary structure with the known exception of *trnS(AGY).* The putative origin of replication of the light strand had the potential to fold into a stem-loop secondary structure and was located between the *trnN* and *trnC* genes in all species, with the exception of *Heleophryne regis* and *Lechriodus melanopyga* (see main text). Three conserved sequence blocks (CSB-1, CSB-2, CSB-3) were identified in the 3' end of the control region of all species. The length of the control regions varied widely among the completely sequenced mt genomes (from 982 bp in *S. thomasseti* to >3866 bp in *T. bolivianus*), and included one or more tandem repeats. The actual length of the mt control regions of two species could not be accurately determined due to the presence of long tandem repeats (200-300 bp in *T. bolivianus* and 1800-2100 bp in *L. melanopyga*), and thus they were estimated from the length of the PCR product in the gel to be 19500-19600bp and 21000-21300 bp, respectively. Notably, the *LTPF* tRNA gene cluster has a 385 bp-long intergenic spacer between *trnP* and *trnF* with no obvious sequence similarity (based on BLAST searches), and it contains an 82 bp-long tandem repeat.

References

1. Benton MJ, Donoghue PCJ: **Paleontological evidence to date the Tree of Life**. *Mol Biol Evol* 2007, **24**:26-53.
2. Roelants K, Gower DJ, Wilkinson M, Loader S, Biju SD, Guillaume K, Moriau L, Bossuyt F: **Global patterns of diversification in the history of modern amphibians**. *Proc Natl Acad Sci USA* 2007, **104**:887-892.
3. Gao K-Q, Shubin NH: **Earliest known crown-group salamanders**. *Nature* 2003, **422**:424-428.
4. San Mauro D: **A multilocus timescale for the origin of extant amphibians**. *Mol Phylogenet Evol* 2010, **56**:554-561.
5. Marjanović D, Laurin M: **Fossils, molecules, divergence times, and the origin of lissamphibians**. *Syst Biol* 2007, **56**:369-388.
6. Rage J, Roček Z: **Redescription of *Triadobatrachus massinoti* (Piveteau, 1936) an anuran amphibian from the early Triassic**. *Palaeontographica Abt A* 1989, **206**:1-16.
7. Ogg JG, Ogg G, Gradstein FM: *The concise geologic time scale*. Cambridge: Cambridge University Press; 2008.
8. Evans SE, Milner AR, Mussett F: **A discoglossid frog from the Middle Jurassic of England**. *Palaeontology* 1990, **33**:299-311.
9. Henrici AC: **A new pipoid anuran from the late Jurasic Morrison formation at Dinosaur National Monument, Utah**. *J Vertebr Paleontol* 1998, **18**:321-332.
10. Martín C, Sanchíz B: Lisanfos KMS. Version 1.2. Online reference [http://www.lisanfos.mncn.csic.es/]. Museo Nacional de Ciencias Naturales, CSIC. Madrid.; 2010.
11. Báez AM: **Tertiary anurans from South America**. In: *Amphibian biology.* Edited by Heatwole H, Carroll RL. Chipping Norton, Australia: Surrey Beatty; 2000: 1388-1401.
12. Boore JL: **Animal mitochondrial genomes**. *Nuc Acids Res* 1999, **27**:1767-1780.
13. Gissi C, Iannelli F, Pesole G: **Evolution of the mitochondrial genome of Metazoa as exemplified by comparison of congeneric species**. *Heredity* 2008, **101**:301-320.
